# Anti-Influenza A Viral Butenolide from *Streptomyces* sp. Smu03 Inhabiting the Intestine of *Elephas maximus*

**DOI:** 10.3390/v10070356

**Published:** 2018-07-05

**Authors:** Fangfang Li, Daiwei Chen, Shengsheng Lu, Guang Yang, Xiaoling Zhang, Zhao Chen, Sheng Fan, Shaohua Wu, Jian He

**Affiliations:** 1 Group of peptides and natural products Research, School of Pharmaceutical Sciences, Southern Medical University, 1838 Guangzhou Avenue North; Guangzhou 510515, China; lff9282@163.com (F.L.); chenday@yeah.net (D.C.); shengshenglu2016@163.com (S.L.); YangG2018@yeah.net (G.Y.); zhangxiaoling@hec.cn (X.Z.); tinycozy@163.com (Z.C.); fan_727@163.com (S.F.); 2 Guangdong Provincial Key Laboratory of Emergency Test for Dangerous Chemicals, Guangdong Provincial Public Laboratory of Analysis and Testing Technology, China National Analytical Center, Guangzhou 510000, China; 3 Key Laboratory for Microbial Resources of the Ministry of Education, Yunnan Institute of Microbiology, School of Life Sciences, Yunnan University, Kunming 650091, China; shwu123@126.com

**Keywords:** anti-influenza A viruses, hemagglutinin, *Streptomyces* sp., butenolide

## Abstract

Actinobacteria are a phylum of bacteria known for their potential in producing structurally diversified natural products that are always associated with a broad range of biological activities. In this paper, using an H5N1 pseudo-typed virus drug screening system combined with a bioassay guided purification approach, an antiviral butanolide (**1**) was identified from the culture broth of *Streptomyces* sp. SMU03, a bacterium isolated from the feces of *Elephas maximus* in Yunnan province, China. This compound displayed broad and potent activity against a panel of influenza viruses including H1N1 and H3N2 subtypes, as well as influenza B virus and clinical isolates with half maximal inhibitory concentration values (IC_50_) in the range of 0.29 to 12 µg/mL. In addition, 1 was also active against oseltamivir-resistant influenza virus strain of A/PR/8/34 with NA-H274Y mutation. Studies on the detailed modes of action suggested that **1** functioned by interfering with the fusogenic process of hemagglutinin (HA) of influenza A virus (IAV), thereby blocking the entry of virus into host cells. Furthermore, the anti-IAV activity of **1** was assessed with infected BALB/c mice, of which the appearance, weight, and histopathological changes in the infected lungs were significantly alleviated compared with the no-drug-treated group. Conclusively, these results provide evidence that natural products derived from microbes residing in animal intestines might be a good source for antiviral drug discovery.

## 1. Introduction

It is well documented that animal bodies, including human beings, are the reservoir of a wide variety of microbes, which play a critical role in the health and welfare of their hosts, and are associated with the etiology and pathogenesis of a large number of diseases [1]. Among the microbes inhabiting the intestinal tract of animals, actinobacteria are a predominant group of bacteria and are known for their potential in producing functional secondary metabolites. In turn, these bioactive metabolites either inhibit the excessive growth of other microorganisms or improve the health of their animal host [2]. Considering the fact that these beneficial molecules inside the animal body have evolved with their host over a long period of time and have been proven to be safe and effective by numerous in vivo “tests”, it is therefore reasonably deduced that the bioactive metabolites isolated from bacteria inside the animal body might possess high potential for use as therapeutic agents, which prompted this investigation of the bioactive components from actinobacteria inhabiting the animal intestinal tract using the bioassay guided approach.

Influenza A viruses (IAVs) are enveloped viruses responsible for seasonal flu epidemics each year. So far, there are two types of drugs available in clinics that target the viral neuraminidase (NA) and the influenza A virus M2 protein. The mechanisms of these drugs involve preventing the virus’ budding from the host cells (oseltamivir and zanamivir) or inhibiting the release of viral RNAs into the cytoplasm of host cells (adamantane derivatives) [3]. However, due to the emergence of drug-resistant viral strains, new and effective therapeutics including new anti-IAV drug targets are urgently needed.

To deal with this challenge, we focused our interest on the hemagglutinin (HA) of influenza A virus by employing it as a possible anti-IAV drug target. As has been reported previously [4], the HA of IAV is a homotrimeric glycoprotein consisting of HA1 and HA2 subunits. In the process of virus entry, the HA1 subunit is responsible for receptor binding [5], while HA2 mediates the viral-endosomal membrane fusion [6,7]. Thus, a conclusion can be drawn that interfering with the function of the HA protein including the HA1 and HA2 subunits would result in the interruption of virus entry and, as a result, lead to prophylactic and therapeutic effects toward IAV infection [8].

Initiated from this point, in our previous work, using the plasmids encoding the HA and NA of A/Thailand/Kan353/2004 with the HIV backbone (pNL4-3.luc.R^−^E^−^), an H5N1 pseudo-typed virus was constructed, by which the potential antiviral compounds were able to be screened and identified by measuring the inhibitory effect toward the infection of H5N1 pseudovirus on Madin-Darby canine kidney (MDCK) cells [9]. We then employed this system to screen the anti-IAV activity of ethyl acetate extracts of the culture broth of actinobacteria from a large number of animal feces samples collected from different areas of China. As a screening result, a bacterial strain, *Streptomyces* sp. Smu03, possessing potent anti-IAV activity was identified, from which a secondary metabolite of butanolide [(4*S*)-4-hydroxy-10-methyl-11-oxo-dodec-2-en-1,4-olide] (**1**) was subsequently purified via a bioassay guided approach. The structure of **1** was determined by the interpretation of spectroscopic data including nuclear magnetic resonance (NMR) and mass spectrometry (MS) spectra, as well as comparison with the literature. In addition, the anti-IAV activity in vitro and in vivo and the mode of action of **1** were investigated. Herein, we report on the isolation, identification, anti-IAV activity and possible mechanism of action of **1**.

## 2. Materials and Methods

### 2.1. Chemicals and Analytical Instruments

The NMR spectra were recorded in deuterated chloroform solution on a Bruker DRX-400 spectrometer (400 and 100 MHz for ^1^H and ^13^C NMR, respectively), and chemical shifts were referenced to tetramethylsilane (TMS) as an internal standard. Electrospray ionization mass spectrometry (ESI-MS) were measured a Waters 3100 single-quadrupole mass spectrometer in the positive mode with a capillary voltage of −3 kV and cone voltage of −30 V. Silica gel (Qingdao Marine Chemical Factory, Qingdao, China), Sephadex LH-20 (Amersham Pharmacia Biotech, Little Chalfont, UK) and reverse-phase silica gel C18 (40–63 μm, Merck, Kenilworth, NJ, USA) were used for the column chromatography. The circular dichroism (CD) data were acquired on a JASCO (J-810) instrument using cuvettes with a 0.2 cm path length.

### 2.2. Cells and Influenza Viral Strains

MDCK cells were cultured in DMEM medium containing 10% fetal bovine serum. Influenza virus including A/FM/1/47 (H1N1) mouse adapted strain, A/Puerto Rico/8/34 (H1N1), A/Puerto Rico/8/34 (H1N1) with NA-H274Y mutation and A/Aichi/2/68 (H3N2), clinical isolates of 690 (H3 subtype), 699 (H3 subtype), and the influenza B virus. All viruses were amplified in 9-day-old embryonated hen eggs and stored at −80 °C. The virus titer was determined by the 50% tissue culture infective dose (TCID_50_) [10].

### 2.3. Fermentation, Extraction and Isolation

The bacterial strain SMU03 was isolated from the feces excreted by adult *Elephas maximus*, collected in Yunnan Province, China, in 2013. The strain was identified to be *Streptomyces* sp. based on its 16S rRNA sequence and morphology characteristics.

A slant culture of the strain was inoculated in a 200 mL Erlenmeyer flask containing 100 mL of seed medium (3% mannitol, 1% glucose, 0.5% yeast extract, 0.1% ammonium succinate, 0.05% K_2_HPO_4_, 0.05% MgSO_4_·7H_2_O, and 0.1 mL of multiple vitamin solution, adjusted to pH 7.5 by 1 M NaOH solution) and incubated at 28 °C for 2 days at 200 rpm. Then, the seed culture was transferred to a 2 L Erlenmeyer flask containing 500 mL of culture medium (1% glucose, 0.3% beef extract, 0.3% peptone, 2% soluble starch, 0.5% yeast extract, 0.3% CaCO_3_, adjusted to pH 7.0 by 1 M NaOH solution) and incubated for 7 days under the same conditions.

The fermentation broth (100 L) was centrifuged at 8000 rpm for 15 min, and the filtrate was absorbed onto D101 macroporous resin sequentially washed with water and 95% EtOH. The fractions eluted with EtOH were collected and evaporated to dryness in vacuo (130.19 g) and partitioned with petroleum ether, dichloromethane (6.46 g), and ethyl acetate (5.05 g). The fraction of dichloromethane was separated by silica gel column chromatography eluting with a gradient of CH_2_Cl_2_ and MeOH to yield 22 fractions. Fraction 6 was further subjected to Sephadex LH-20 eluting with MeOH to produce 8 subfractions (Fr.1~Fr.8). Compound **1** (13 mg), the active compound, was chromatographed by reversed-phase silica gel C_18_ (60% aqueous MeOH).

### 2.4. Structure of (4S)-4-Hydroxy-10-methyl-11-oxo-dodec-2-en-1,4-olide (***1***)

Colorless oil, 1H NMR (CDCl_3_, 400Hz) δ: 7.44 (1H, d, *J* = 5.7 Hz, H-3), 6.10 (1H, d, *J* = 5.6 Hz, H-2), 5.03 (1H, t, *J* = 6.5, 7.0 Hz, H-4), 2.49 (1H, m, H-10), 2.13 (3H, s, H-13), 1.76 (1H, m, H-5), 1.63 (1H, m, H-9), 1.60 (1H, m, H-5), 1.43 (2H, m, H-6), 1.32 (3H, m, H-7 and H-9), 1.27 (2H, m, H-8), 1.07 (3H, d, *J* = 6.9 Hz, H-13); 13C NMR (CDCl_3_, 100 Hz) δ: 212.6 (s, C-11), 172.9 (s, C-1), 156.0 (d, C-3), 121.4 (d, C-2), 83.1 (d, C-4), 46.9 (d, C-10), 33.3 (t, C-9), 32.9 (t, C-5), 29.1 (t, C-7), 27.8 (q, C-12), 26.8 (t, C-8), 24.7 (t, C-6), 16.1 (q, C-13). ESI-MS *m/z* 225 [M + H]^+^. CD ([θ]205, MeOH): +24,800. These data are the same as the data reported in the literature [11,12].

### 2.5. Cytotoxic Assay

The cytotoxicity of compound **1** was determined by the MTT (3-[4,5-dimethyl thiazol-2-yl]-2,5-diphenyl tetrazolium bromide) assay as described previously [13] with some modifications. Briefly, MDCK cells were prepared in 96-well plates (1 × 10^4^ cells/well) and when the cell monolayer was confluent, the cells were incubated with 200 μL/well of various concentrations of the compound for 48 h. Then, cells were incubated with 100 μL MTT (0.5 mg/mL) in DMEM at 37 °C for 4 h and treated with 150 μL DMSO (dimethyl sulfoxide). The absorbance was read at 570 nm using a microtiter plate reader (Genios Pro Tecan, Mānnedorf, Switzerland).

### 2.6. Cytopathic Effect (CPE) Reduction Assay

MDCK cells were cultured in 96-well plates (2 × 10^4^ cells/well) for 24 h. A series of two-fold diluted compound **1** solutions was pre-incubated with virus (100 TCID_50_) at 37 °C for 30 min, and the cells were incubated with the virus-compound mixtures for 1 h after washing twice with PBS. Then replace it with serum-free DMEM containing 1 mg/mL TPCK-trypsin. At 48 h post-infection, cell viability was determined using the previously described MTT assay. The experiment was independently repeated at least three times with arbidol as a positive control.

### 2.7. CPE Reduction Assay with Different Drug Administrations

To examine the mechanism underlying viral inhibition by compound **1**, four different time points for drug administration were utilized in our experiments. In brief, MDCK cells were seeded into 96-well plates. Then, the cells or viruses were respectively treated as follows: (1) pretreatment to cell: compound **1** were pre-incubated with cells for 30 min at 37 °C, and then compound **1** was removed before adsorption of influenza virus A/PR/8/34 (H1N1) (100 TCID_50_); (2) pretreatment to virus: virus was pre-incubated with compound **1** before adding virus-compound mixtures to cells; (3) during infection: compound **1** were added to the cells with influenza virus at the same time; and (4) after infection: compound **1** was added to the cells after adsorption of influenza virus to the cells. After infection for 1 h, the cells were washed and cultured in serum-free DMEM containing 1 mg/mL TPCK-trypsin, followed by incubation for 48 h. In this experiment, arbidol was set as a positive control. The antiviral effect was assessed using an MTT assay. The experiment was repeated at least three times.

### 2.8. Quantitative Real-Time Polymerase Chain Reaction (PCR) Assay

Approximately 90% of MDCK cells were confluent with influenza A/PR/8/34 (H1N1) virus infected at 100 TCID_50_ pretreated with compound **1**. Trizol Reagent (Sigma, St. Louis, MO, USA) was used to extract the total RNA, and the cDNA was reverse transcribed using the original RT Master Mix kit (Takara, Beijing, China) according to the instructions. Real-time quantitative polymerase chain reaction (qPCR) was performed by ABI 7500 Sequence Detection System (Applied Biosystems, Foster City, CA, USA). Each sample was tested at least three times independently. The 2^−ΔΔ*C*t^ method was used to compare the relative content of the *HA* gene. The primer sequences used for the influenza A matrix genes are 5′ -ATGAGYCTTYTAACCGAGGTCGAAACG-3′ (forward), 5′-TGGACAAANCGTCTACGCTGCAG-3′ (reverse), and the sequences for the *GAPDH* gene are 5′-AGGGCAATGCCAGCCCCAGCG-3′ (forward), 5′-AGGCGTCGGAGGGCCCCCTC-3′ (reverse).

### 2.9. Fluorescence Confocal Microscopy Assay

The MDCK cells were inoculated into a special confocal dish containing 1 × 10^3^ cells per well and cultured overnight. After the cells grew to 60%, methanol (−20 °C precool) was added to each well of fixed cells, and 20 min later, 1 mL of 4% BSA was added and stored at 37 °C for 20 min. Then, the supernatant was removed and incubated with NP primary antibodies (1:250 dilution, Santa Cruz, CA, USA) overnight at 4 °C. After discarding the supernatant, FITC-labeled secondary antibody (diluted 1:250) was added for 4 h at 37 °C, and then MDCK cells stained with Diamidine phenylindole (DAPI) solution for 10 min. The results observed by confocal immunofluorescence microscopy (Olympus, Tokyo, Japan).

### 2.10. Neuraminidase (NA) Inhibition Assay

The NA inhibitory activity of the compound was tested by measuring the intensity of the fluorescence that produced by the neuraminidase cleave the MU-NANA substrate, as reported previously [14]. Briefly, A/PR/8/34 (H1N1) virus was pre-incubated with compound **1** in 33 mM morpholine ethylsulfonic acid (MES) buffer (containing 4 mM CaCl_2_, pH 6.5) at room temperature for 45 min, then 50 µL of MU-NANA dissolved in 33 mM MES buffer was added to each well. The plate was incubated for another 1 h at 37 °C in the dark and terminated with 100 μL of 34 mM NaOH (containing 83% ethanol). Fluorescence was measured at 340 nm for excitation wavelength and 440 nm for emission wavelength. Oseltamivir and zanamivir in 33 mM MES buffer as a positive control. The inhibition rate of NA activity was calculated using the following formula:

Inhibition rate (%) = (Fvirus − Fsample)/(Fvirus − Fsubstrate) × 100%

### 2.11. Hemolysis Inhibition Assay

A hemolysis inhibitory assay was applied to determine the hemolytic inhibitory effect of the extracts on virus-induced hemolysis at specific pH. Briefly, 100 µL of compound **1** diluted in PBS was mixed with an equal volume of influenza virus A/PR/8/34 (H1N1) (10^6^ TCID_50_/0.1 mL) at room temperature for 30 min. Then, 200 µL of 2% chicken erythrocytes (37 °C preheat) was added and incubated at 37 °C for 30 min. To trigger HA acidification and hemolysis, 100 µL of sodium acetate (0.5 M, pH 5.03) was added and mixed well and incubated at 37 °C for 30 min. The plates were centrifuged at 3000 rpm for 10 min, 300 µL of supernatant was transferred to a new 96-well plate, and the absorbance at 535 nm was read.

### 2.12. Polykaryon Formation Inhibition Assay

Using the following previously reported procedure [15], the fusion inhibitory effect of compound **1** was further assessed. Briefly, MDCK cells (2 × 10^5^ cells per well; 12-well plate) were transfected with the plasmid encoding the HA of A/Thailand/Kan353/2004 (0.8 µg/well) by using transfection reagent of polyetherimide (PEI). After 8 h, the transfection medium was replaced by complete medium. 48 h later, the cells were treated with tosyl-phenylalanine chloromethyl-ketone (TPCK)-treated trypsin (5 µg/mL) for 15 min at 37 °C. After that, they were rinsed with PBS twice and pretreated with the compound **1** for 15 min at 37 °C. Then, the cells were incubated with acidic buffer (DMEM, pH 5.0) containing the compound **1** for another 15 min at 37 °C. After the reaction, cells were washed with PBS, and then incubated with complete medium for 3 h at 37 °C to allow the formation of polykaryons. Finally, the cells were fixed with methanol, stained with Giemsa, and examined by microscopy at 200× magnification. The syncytium formation was quantified by counting the number of polykaryons (containing five or more nuclei) in 10 random fields of the plate.

### 2.13. In Vivo Protective Efficacy in Mice

By adopting the protocol as reported previously [16], five- to seven-week-old (20–23 g) specific pathogen free (SPF) male BALB/c mice were purchased from the animal experiment center of southern medical university and randomly divided into 5 groups (5 mice in each group): blank control group; virus control group; positive control group (arbidol at a dose of 10 mg/kg); compound **1** high dose group (1 mg/kg); and compound **1** low dose group (0.5 mg/kg), all drugs were dissolved in 0.1% methanol-PBS. On the first day, each group of mice was infected with the virus in the morning and afternoon except for the blank control group. The infection method was to infect 50 μL of A/FM/1/47 virus solution with a TCID_50_ value of about 10^5^ into each mouse’s nasal cavity, and intragastric administration occurred immediately after infection with the same amount of saline instead of drugs in the virus control group and blank control group. Drugs were given only once in the next two days. On the fourth day, all mice were executed, and organs such as the lung were collected and weighed. The lung index is expressed as the ratio of the average lung weight to the average body weight. After washing twice with 0.9% saline, the lungs were fixed in freshly prepared 37% formalin for 24 h. After that, HE staining of pathological sections was performed to observe the degree of pulmonary virus infection. The operation of animal experiments was performed by the Institutional Animal Care and Use Committee (IACUC) with approval ID I-IACUC2016002 (The approval date is January 2016). The feeding and experiment process were carried out in the P3 level laboratory of Guangdong experimental animal monitoring.

### 2.14. Statistical Analysis

Graph Pad Prism 5 (San Diego, CA, USA) software was used to calculate the half cytotoxic concentration (CC_50_) and the half inhibitory concentration (IC_50_) values. Each experiment was repeated at least 3 times and is represented as the means ± standard deviation (SD). Data were analyzed with the one-way analysis of variance (ANOVA) method by using SPSS20.0 software (IBM, Armonk, NY, USA). The significance was defined as * *p* < 0.05, ** *p* < 0.01, *** *p* < 0.001.

## 3. Results

### 3.1. A Butenolide Was Isolated from Streptomyces sp. Smu03 Residing in the Intestine of Elephas maximus

**Figure 1:**
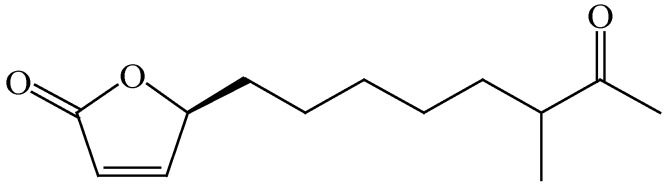
The structure of compound **1**.

By employing H5N1 pseudo-typed virus as the drug screening system, the ethyl acetate extracts from the culture broth of a bacterial strain, *Streptomyces* sp. Smu03, possessing a potent anti-IAV activity was identified. *Streptomyces* sp. Smu03 was isolated from the feces of an adult Asian elephant, *Elephas maximus*, in Yunnan province, China, and the anti-IAV activity was confirmed by re-test with influenza A/PR/8/34 (H1N1) virus. Subsequently, the bioassay guided purification approach was carried out from the culture broth of this strain. As a result, a bioactive butenolide (**1**), (4*S*)-4-hydroxy-10-methyl-11-oxo-dodec-2-en-1,4-olide, was isolated and identified by extensive analyses of the spectroscopic data including NMR, MS and CD spectra, as well as in comparison with the literature [12] (Figure 1). The butenolides are often isolated from the secondary metabolites of fungi, bacteria, and gorgonians [11,17,18], which are known for their potential in the promotion of metabolite production and spore formation [19,20].

### 3.2. Butenolide ***1*** is Active against a Wide Variety of Influenza A Viruses and has Moderate Cellular Toxicity Toward MDCK Cells

Cytopathic effect (CPE) inhibition assays were used to evaluate the anti-IAV activities of **1**. The viral strains were influenza A/PR/8/34 (H1N1), A/Aichi/2/68 (H3N2), A/FM/1/47 (H1N1) mouse adapted strains, and oseltamivir-resistant influenza virus strains of A/PR/8/34 with NA-H274Y mutation. As shown in Table 1, compound **1** was active against all the tested strains with IC_50_ values ranging from 0.29 to 12 µg/mL, close to the positive control of arbidol. To confirm the anti-viral activity of **1**, the three clinical isolates 690 (H3), 699 (H3) and influenza B viruses were tested as well. As expected, **1** also displayed activity against these clinical isolates. In addition, **1** showed more activity toward influenza A/Puerto Rico/8/34 viral strain than other influenza viral strains.

**Figure 2:**
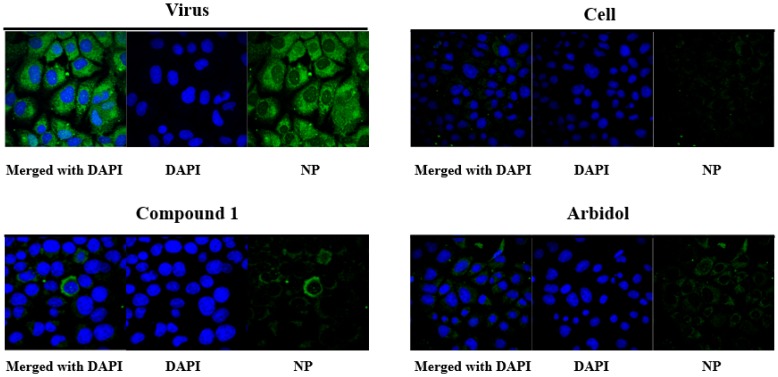
Anti-influenza A virus effect of compound **1** visualized by confocal microscopy (200×). “Virus” refers to the addition of influenza A virus into MDCK cells, while “Cell” refers to MDCK cells without the addition of virus. Viral strain: influenza A/PR/8/34 (H1N1) virus.

The confocal microscopy with NP-specific antibodies was then employed to confirm the anti-IAV activity of **1** on influenza virus. As indicated in Figure 2, at 24 h post-infection, the NP protein localized in the cytoplasm was observed (“Virus” vs. “Cell”, where “Cell” refers to MDCK cells without addition of virus). In contrast, the NP protein signals declined in the presence of **1** (10 µg/mL) or the positive control arbidol (10 µg/mL) by using the pre-treatment method as used in the CPE assay, which was in agreement with the anti-IAV effects observed from the CPE assay, as well as reported from the literature [21] (Table 1).

Next, an MTT assay was applied to evaluate the cellular toxicity of **1** [22]. As shown in Table 1, the CC_50_ value of **1** toward MDCK cells was moderately toxic at 38.07 ± 1.50 µg/mL and lower than the positive drug arbidol (12.97 ± 2.25 µg/mL), indicating that **1** has supportive selectivity indices in the development of an anti-IAV agent.

### 3.3. Butenolide ***1*** Displays the Anti-Influenza A Virus (IAV) Effect in the Early Stage of Infection

**Figure 3:**
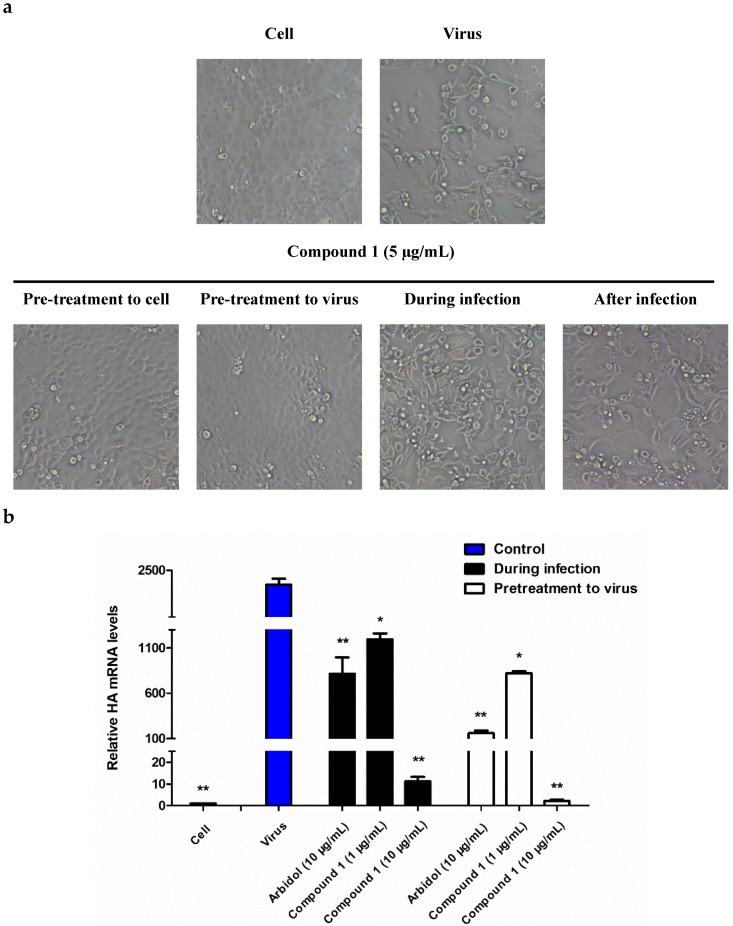
The anti-IAV activity tested with different drug administration approaches. (**a**) Cytopathic effects of H1N1-infected MDCK cells with different drug treatment approaches (100×, the concentration of **1** was 10 µg/mL). (**b**) The antiviral effects of **1** against influenza A/PR/8/34 (H1N1) evaluated by measuring the mRNA level of the *HA* gene after treatment with **1** with two drug administration approaches, namely, “pretreatment to virus” or “during infection”. Statistical significance of the data in the virus group was defined as * *p* < 0.05, ** *p* < 0.01, *** *p* < 0.001 with the one-way analysis of variance (ANOVA) method.

To study the possible mechanism and detailed inhibitory step of **1** on the influenza virus life cycle, we tested the anti-viral effect of **1** toward influenza A/PR/8/34 (H1N1) virus using four different time points for drug administration: preventive treatment, pre-incubation treatment, simultaneous treatment, and post-treatment, as described before [14]. The results were then observed under a microscope as shown in Figure 3a and further quantitatively measured by the CPE assay as shown in Table 2. As a result, the pre-incubation treatment of virus was shown to be the most active drug administration compared with the other approaches, indicating that **1** functioned in the early stage of the influenza viral life cycle.

The early stage inhibitory effect of **1** on the influenza A viral life cycle was further assessed by measuring the mRNA level of the hemagglutinin gene (HA) of influenza A virus. As demonstrated in Figure 3b, at 24 h post-infection, an apparently decreased mRNA level of HA was observed. In addition, the difference in the RT-PCR level of the “pretreatment to virus” and “during infection” drug administration supported the observation obtained from the CPE assay (Figure 3b).

### 3.4. Butenolide ***1*** is Inactive toward the Enzymatic Activity of NA

**Figure 4:**
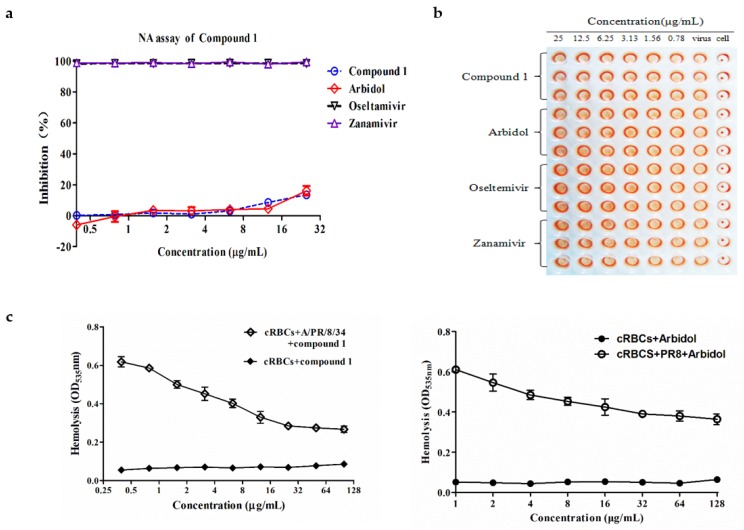
(**a**) Neuraminidase (NA) inhibition assay. (**b**) The hemagglutination inhibition (HI) assay of compound **1**. (**c**) Inhibition assay of HA-mediated hemolysis of chicken erythrocytes.

Two enveloped proteins of influenza A virus are presented in the viral particle surface: HA and NA. The NA inhibition assay was performed to test whether NA is the possible target of **1**. By adopting the same protocol as described previously [14], the data in Figure 4a showed that, in comparison with the positive control of oseltamivir and zanamivir, no significant inhibitory effect was observed for **1** in the test range of 0.39 to 25 µg/mL, indicating that NA was not the possible target of **1**.

### 3.5. The HA2 Subunit of Hemagglutinin (HA) is the Possible Target of Butenolide ***1***

The surface glycoprotein HA is composed of HA1 and HA2 two subunits, both of which play a crucial role in mediating the entry of virus into host cells [23]. Therefore, we studied the interactions between HA and **1** in which we explored the possible target of **1**. In the first step, the HA inhibition (HI) assay was performed [24] and the data in Figure 4b showed that no apparent inhibition on agglutination of chicken erythrocytes was observable in the test range, thus indicating that the sialic acid binding site on HA1 was not the possible target of **1**.

The hemolysis inhibition assay was then used to test whether the HA2 subunit was the possible target of **1**. By following a protocol as reported previously [25], the assay in Figure 4c showed that, in the presence of **1**, the hemolytic effect was reduced under the acidic condition compared with the virus control, which was similar to the hemolytic effect of the positive control of arbidol, indicating that the anti-IAV effect of **1** possibly resulted from interaction with the HA2 subunit, which interrupted the fusogenic process of membrane fusion [26].

**Figure 5:**
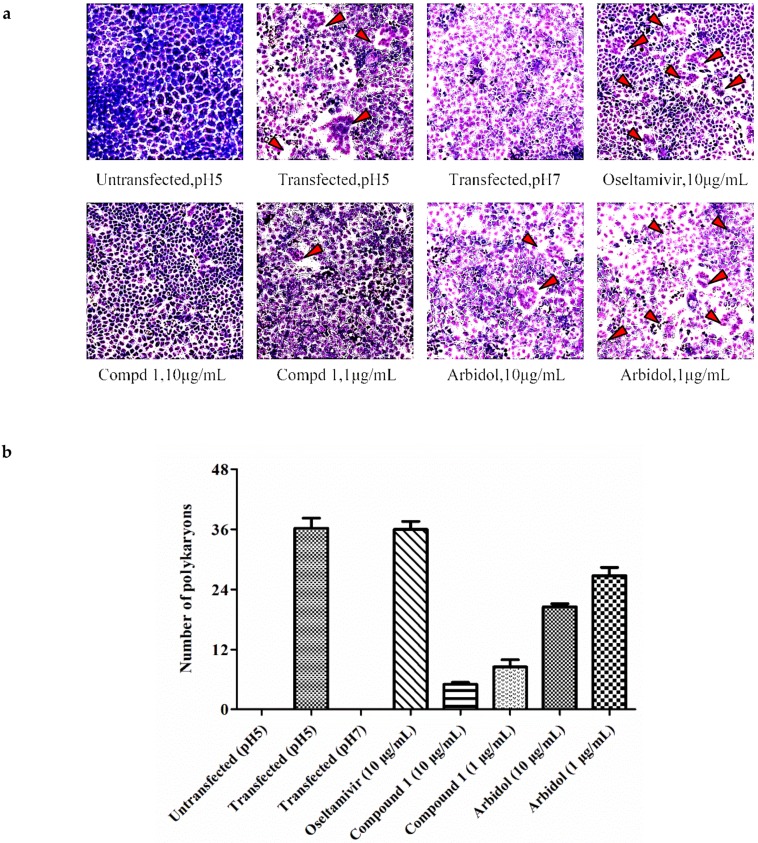
(**a**) Polykaryon formation inhibition assay (100×). (**b**) Syncytium formation was quantified by counting the number of polykaryons.

To confirm the fusion inhibitory effect of **1**, the polykaryon inhibition assay was carried out next. As shown in Figure 5, the MDCK cells expressing HA were mixed with either 1% methanol or each tested compound, including **1**, arbidol and oseltamivir, and then acidified to pH 5.0 to initiate the formation of polykaryons that resulted from the membrane fusion. The data in Figure 5 showed that **1** and arbidol inhibited syncytium formation compared with the cells treated with 1% methanol and oseltamivir, which provided additional support for the fusogenic inhibitory effect of **1**.

### 3.6. Butenolide ***1*** is Effective against IAV In Vivo

Due to the limited amount of **1**, the preliminary protective effect of **1** was further assessed in vivo by testing influenza A virus-infected mice. The experiment protocol was adopted as reported previously by using 5- to 7-week-old male Balb/c mice [16], to which 10^5^ TCID_50_ of influenza A/FM/1/47 (H1N1) virus was intranasally inoculated twice in a volume of 50 µL of normal saline (NS). Subsequently, the mice were orally administrated with **1** or arbidol after virus exposure. As such, the total amount of compound was given twice in the first day of infection and then once a day in the following two post-infection days before the mice were sacrificed for the next step. The anti-IAV efficacy of **1** was then evaluated based on the appearance, weight and histopathological changes in the lungs of the sacrified mice.

**Figure 6:**
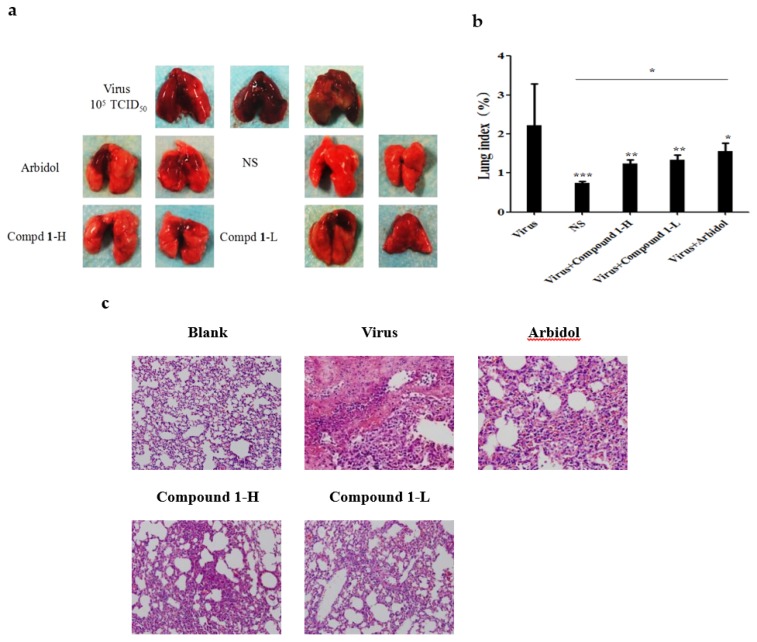
Microscopic appearance (**a**), lung index (**b**) and histopathological (200×) (**c**) changes in the lungs of FM1-infected mice. The lung index is expressed as the ratio of the average lung weight to the average body weight. Mice were intranasally inoculated with FM1 virus and sacrificed three days post-infection. Arbidol at a dose of 10 mg/kg; compound **1** high dose group (1 mg/kg) and low dose group (0.5 mg/kg). Statistical significance of the virus group was defined as * *p* < 0.05, ** *p* < 0.01, *** *p* < 0.001 with the one-way ANOVA method).

As a result, the histopathological changes (Figure 6c) in the lungs clearly showed the difference between the virus-infected and normal mice, of which a large amount of alveolar collapse around bronchioles was observed in virus-infected mice, while it was dramatically decreased in the compound-treated group (Figure 6c), which was consistent with the phenomena observed by Yamashita et al [26].

## 4. Discussion

IAVs are one of the main pathogens responsible for seasonal epidemics, widespread morbidity and mortality in humans [27]. To date, compared with the rapid increase in drug-resistant influenza viral strains, the availability of clinically used drugs is limited, which represents a compelling pressure for novel antiviral drugs. Natural products have long been recognized as a rich source for drug discovery [28], and 34% of new medicines approved by the US Food and Drug Administration (FDA) between 1981 and 2010 were natural prod­ucts or directly derived from natural products [29,30], thus suggesting a promising source for the discovery of new anti-IAV drugs. In this study, based on H5N1 pseudo-typed viruses [31], the EtOAc extracts obtained from the culture broth of a large number of actinobacterial strains isolated from animal feces were screened. As a result, a butanolide [(4*S*)-4-hydroxy-10-methyl-11-oxo-dodec-2-en-1,4-olide] (**1**) was isolated and identified from the bacterium of *Streptomyces* sp. Smu03 residing in the intestine of *Elephas maximus*.

The anti-IAV activity test indicated that **1** exhibited a broad antiviral activity against a panel of influenza A viruses with an IC_50_ ranging from 0.29 to 12 µg/mL, of which the preventive treatment was proven to be the most effective drug administration (Figure 3), suggesting that **1** functioned by interfering with the early stage of the life cycle of influenza A virus. Therefore, it can be deduced that HA should be a key enzyme and play a significant role in viral entry, as indicated by numerous studies [13,32,33].

HA is composed of the two subunits HA1 and HA2; therefore, we tested the receptor binding inhibitory activity and fusion inhibitory activity of **1** by employing HA inhibition (HI) and hemolysis inhibition assays. As shown in Figure 4b,c, compound **1** was unable to block the absorption of virus into targeting cells while exhibiting apparent inhibitory effects on the fusion of virus-host cell membrane under acidic conditions, indicating that the possible target of **1** should be the HA2 subunit of HA, which was further supported by the polykaryon formation inhibition assay (Figure 5).

The preliminary in vivo anti-IAV activity of **1** was assessed by the test with infected Balb/c mice. The inspection of the appearance, weight and histopathological changes in the infected lungs suggested that significant alleviation was achieved after treatment with **1** compared with the untreated group (Figure 6), thus confirming the anti-viral potential of **1**.

Conclusively, in this paper, we describe the anti-IAV activity and possible modes of action of butenolide (**1**). The data showed that **1** not only possesses a broad and potent anti-IAV activity in vitro and in vivo but also exhibits different modes of action from the currently used drugs. As indicated in the mechanism study, the anti-IAV effect of **1** resulted from interactions with the HA2 subunit by preventing the fusogenic process of the viral-host cell membrane under acidic conditions. Notably, this is the first study to identify an anti-IAV compound as well as to investigate the possible mechanism from the bacteria residing in the intestine of an animal. In addition to the virus entry blocking effect of **1**, we also observed (Figure 3a and Table 2) that the interaction with host cells might have additional effects on anti-IAV activity; therefore, we believe that **1** might play multiple protective roles on its host body in addition to inhibiting the entry of the virus, about which more extensive investigations are needed to support this observation.

## Figures and Tables

**Table 1:** The anti-influenza viral activity and cytotoxicity of **1** (µg/mL).

Name	IC_50_ ± SD *^a^*							CC_50_ ± SD *^b^*
	H3N2 *^c^*	H1N1 *^d^*	H1N1 *^e^*	H1N1 *^f^*	H3 (690) *^g^*	H3 (699)	B	
**1**	7.59 ± 0.72	6.10 ± 1.30	0.29 ± 0.06	3.60 ± 0.43	5.45 ± 1.83	4.59 ± 0.43	11.69 ± 0.92	38.07 ± 1.50
Arbidol	>10	5.58 ± 0.69	0.45 ± 0.16	NT *^h^*	6.51 ± 170	3.85 ± 0.70	24.58 ± 0.28	12.97 ± 2.25

*^a^* the anti-influenza A virus (IAV) activity was determined with the cytopathic effect (CPE) assay by the pretreatment approach; *^b^* cytotoxicity against MDCK cells was tested by the MTT assay; *^c^* influenza A/Aichi/2/68 viral strain; *^d^* A/FM/1/47 mice adapted viral strain; *^e^* A/Puerto Rico/8/34; *^f^* influenza A/Puerto Rico/8/34 virus with NA-H274Y mutation. *^g^* clinical isolates: 690(H3), 699(H3) and B viruses; *^h^* not tested.

**Table 2:** The anti-influenza A viral activity of **1** in various administration time *.

Name	IC_50_ ± SD (µg/mL)			
	Pretreatment to Cell	Pretreatment to Virus	During Infection	After Infection
Compound **1**	0.96 ± 0.10	0.29 ± 0.06	7.50 ± 0.59	>25
Arbidol	>25	0.45 ± 0.16	6.80 ± 1.45	>25

* Influenza A/Puerto Rico/8/34 virus.
